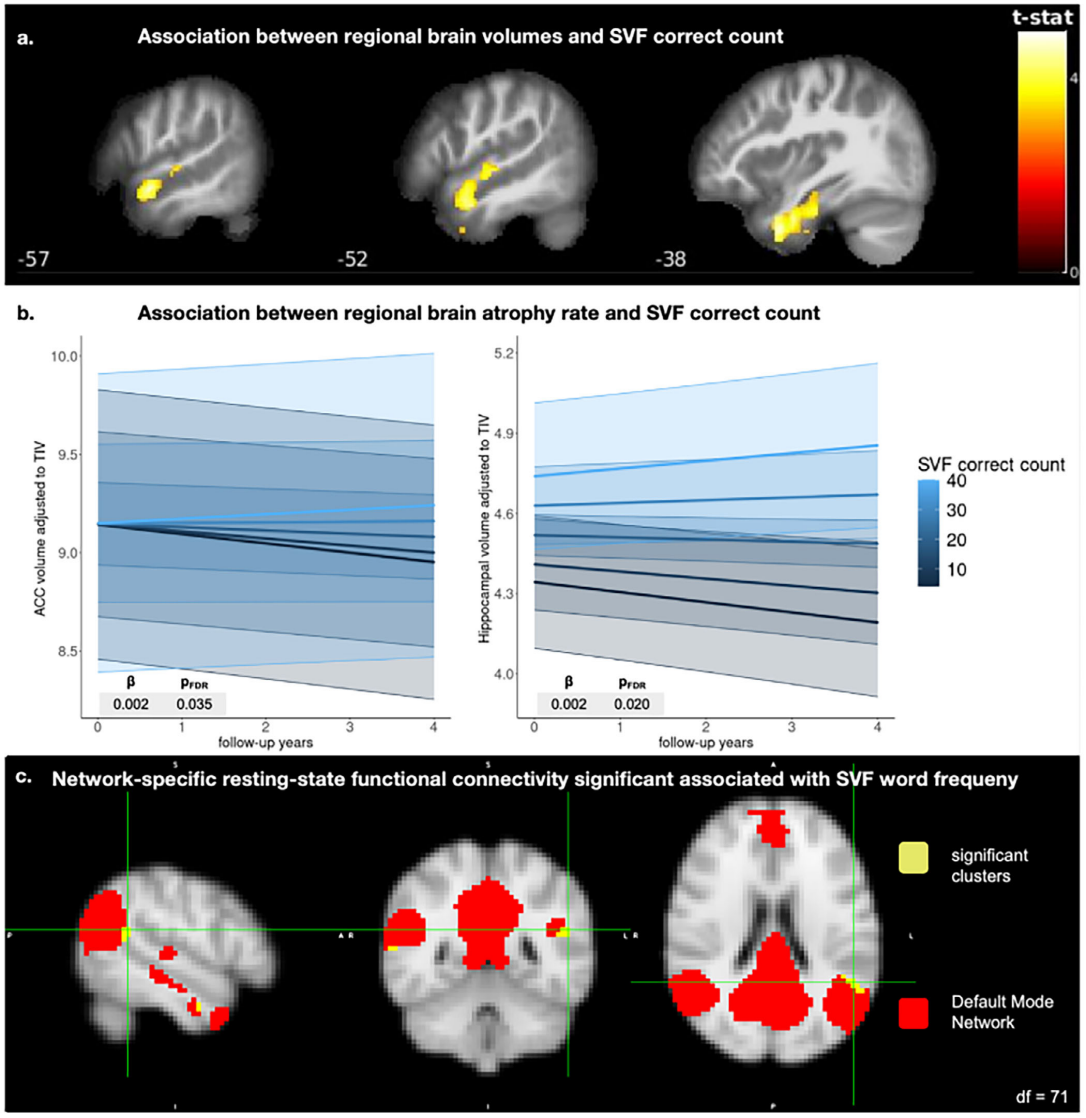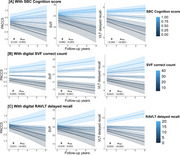# Leveraging Digital Speech Features for Early Identification of Alzheimer's Disease: Associations with structural and functional brain changes over time

**DOI:** 10.1002/alz70857_098534

**Published:** 2025-12-24

**Authors:** Qingyue Li, Stefanie Köhler, Alexandra König, Martin Dyrba, Zampeta‐Sofia Alexopoulou, Elisa Mallick, Nicklas Linz, Anja Schneider, Annika Spottke, Björn Falkenburger, Christoph Laske, Emrah Düzel, Frank Jessen, Inga Zerr, Jens Wiltfang, Josef Priller, Luca Kleineidam, Melina Stark, Michael Wagner, Gabor C Petzold, Fedor Levin, Stefan Teipel

**Affiliations:** ^1^ Rostock University Medical Center, Rostock, Germany; ^2^ German Center for Neurodegenerative Diseases (DZNE), Rostock, Germany; ^3^ ki:elements GmbH, Saarbrücken, Germany; ^4^ CoBTeK (Cognition Behaviour Technology) Research Lab, University Côte d’azur, Nice, France; ^5^ Department of Neurodegenerative Diseases and Geriatric Psychiatry, University of Bonn Medical Center, Bonn, Germany; ^6^ German Center for Neurodegenerative Diseases (DZNE), Bonn, Germany; ^7^ University of Bonn, Bonn, Germany; ^8^ German Center for Neurodegenerative Diseases (DZNE), Venusberg‐Campus 1, 53127, Bonn, Germany; ^9^ University Hospital Carl Gustav Carus, Technische Universität Dresden, Dresden, Germany; ^10^ German Center for Neurodegenerative Diseases (DZNE), Dresden, Germany; ^11^ German Center for Neurodegenerative Diseases (DZNE), Tübingen, Germany; ^12^ Section for Dementia Research, Hertie Institute for Clinical Brain Research and Department of Psychiatry and Psychotherapy, University of Tübingen, Tübingen, Germany; ^13^ German Center for Neurodegenerative Diseases (DZNE), Magdeburg, Germany; ^14^ Institute of Cognitive Neurology and Dementia Research (IKND), Otto‐von‐Guericke University, Magdeburg, Sachsen Anhalt, Germany; ^15^ Faculty of Medicine and University Hospital Cologne, University of Cologne, Cologne, Germany; ^16^ Excellence Cluster on Cellular Stress Responses in Aging‐Associated Diseases (CECAD), University of Cologne, Cologne, Germany; ^17^ University Medical Center, Georg August University, Goettingen, Germany; ^18^ German Center for Neurodegenerative Diseases (DZNE), Goettingen, Germany; ^19^ German Center for Neurodegenerative Diseases (DZNE), Göttingen, Germany; ^20^ Neurosciences and Signaling Group, Institute of Biomedicine (iBiMED), Department of Medical Sciences, University of Aveiro, Aveiro, Portugal; ^21^ University Medical Center Goettingen (UMG), Goettingen, Germany; ^22^ Department of Psychiatry and Psychotherapy, Technical University of Munich, Munich, Germany; ^23^ University of Edinburgh and UK DRI, Edinburgh, United Kingdom; ^24^ Charité – Universitätsmedizin Berlin, Berlin, Germany; ^25^ German Center for Neurodegenerative Diseases (DZNE), Berlin, Germany; ^26^ German Center for Neurodegenerative Diseases (DZNE), Bonn, NRW, Germany; ^27^ Division of Vascular Neurology, Department of Neurology, University Hospital Bonn, Bonn, NRW, Germany

## Abstract

**Background:**

Speech features extracted from automated remote cognitive assessments correlate with performance on traditional cognitive tasks in individuals at risk of Alzheimer's Disease (AD), demonstrating their potential to support early diagnosis. However, the capability of these features to signal early AD‐related brain changes remains less explored.

**Method:**

Within the PROSPECT‐AD study, 234 participants ranging from cognitively normal to mild cognitive impairment were recruited from the German DZNE longitudinal cohorts DELCODE and DESCRIBE. At home, all participants completed the phone‐based and chatbot‐guided Semantic Verbal Fluency task (SVF) and the Rey Auditory Verbal Learning Test (RAVLT). Linguistic and acoustic features were automatically extracted from phone call recordings using an AI model to calculate task‐specific and composite cognitive scores. Structural MRI, functional MRI, and various paper‐and‐pencil cognitive scores were collected during cohort visits. We employed multiple linear regression, mixed‐effects models, and independent component analysis (ICA), followed by voxel‐wise post hoc analyses, to assess associations between digital speech‐based indicators and: (1) cross‐sectional brain atrophy (*n* = 108), (2) longitudinal brain atrophy (*n* = 90), (3) cross‐sectional resting‐state functional connectivity (*n* = 86), and additionally (4) trajectories of cognitive decline (*n* = 146).

**Result:**

SVF correct counts were positively associated with brain volumes in the left temporal pole, left inferior, middle, and superior temporal gyri (The t_(100)_ values ranged from 4.48 to 4.96) in voxel‐wise analyses (Figure 1a). Longitudinal analyses indicated that higher SVF correct counts were linked to slower rates of hippocampal and anterior cingulate atrophy (Figure 1b). Functional connectivity analyses suggested that SVF features, such as word frequency, were associated with rsFC areas within the default mode network (The t_(71)_ values ranged from 3.65 to 3.85) (Figure 1c). Higher composite cognitive scores, along with SVF and RAVLT features, were associated with slower cognitive decline, as measured by established paper‐and‐pencil cognitive assessments, including the Preclinical Alzheimer Cognitive Composite (PACC) 5, SVF, and RAVLT delayed recall (Figure 2).

**Conclusion:**

Phone‐based cognitive assessments hold promise as a remote and scalable tool for identifying AD‐related structural and functional brain changes. They offer predictive value for cognitive trajectories in pre‐dementia populations. This approach could aid in identifying individuals at risk while guiding further evaluation, broadening their utility beyond cognitive screening.